# Diagnostic methods and therapeutic options of uveal melanoma with emphasis on MR imaging—Part II: treatment indications and complications

**DOI:** 10.1186/s13244-021-01001-w

**Published:** 2021-06-04

**Authors:** Pietro Valerio Foti, Mario Travali, Renato Farina, Stefano Palmucci, Corrado Spatola, Rocco Luca Emanuele Liardo, Roberto Milazzotto, Luigi Raffaele, Vincenzo Salamone, Rosario Caltabiano, Giuseppe Broggi, Lidia Puzzo, Andrea Russo, Michele Reibaldi, Antonio Longo, Paolo Vigneri, Teresio Avitabile, Giovani Carlo Ettorre, Antonio Basile

**Affiliations:** 1 Department of Medical Surgical Sciences and Advanced Technologies “G.F. Ingrassia” – Radiology I Unit, University Hospital Policlinico “G. Rodolico-San Marco”, Via Santa Sofia, 78 – 95123 Catania, Italy; 2 Department of Medical Surgical Sciences and Advanced Technologies “G.F. Ingrassia” – Section of Anatomic Pathology, University of Catania, Via Santa Sofia, 78 – 95123 Catania, Italy; 3 Department of Ophthalmology, University of Catania, Via Santa Sofia, 78 – 95123 Catania, Italy; 4 Department of Clinical and Experimental Medicine, Center of Experimental Oncology and Hematology, University Hospital Policlinico “G. Rodolico-San Marco”, Via Santa Sofia, 78 – 95123 Catania, Italy

**Keywords:** Magnetic resonance imaging, Melanoma, Eye, Brachytherapy, Proton therapy

## Abstract

Therapy of uveal melanoma aims to preserve the eye and its function and to avoid metastatic dissemination. The treatment choice is difficult and must keep into account several factors; the therapeutic strategy of uveal melanoma should therefore be personalized, sometimes requiring to combine different treatment techniques. Nowadays globe-sparing radiotherapy techniques are often preferred to enucleation. Plaque brachytherapy, the most commonly used eye-preserving therapy, is suitable for small- and medium-sized uveal melanomas. Proton beam radiotherapy is indicated for tumours with noticeable size, challenging shape and location, but is more expensive and less available than brachytherapy. Enucleation is currently restricted to advanced tumours, uveal melanomas with orbital or optic nerve involvement, blind and painful eyes because of treatment-related complications (neovascular glaucoma, chronic inflammatory processes). The effect of proton beam therapy on neoplastic tissue is related to direct cytotoxic action of the radiations, impairment of neoplastic vascular supply and immunologic response. Complications after radiotherapy are frequent and numerous and mainly related to tumour thickness, radiation dose and distance between the tumour and optic nerve. The purpose of this pictorial review is to provide the radiologists with awareness about diagnostic methods and therapeutic options of uveal melanoma. In the present second section, we discuss the therapeutic management of uveal melanoma, describing the main ocular-conserving radiotherapic techniques. We subsequently present an overview of the effects of radiations on neoplastic tissue. Lastly, we review ocular complications following radiotherapy that should be evaluated by radiologists during follow-up MRI examinations.

## Key points

- Plaque brachytherapy is suitable for small- and medium-sized uveal melanomas.
- Proton beam radiotherapy is feasible for large-sized tumors with challenging location.
- Enucleation is indicated for advanced melanomas and painful eyes because of complications.
- Radiation-induced necrosis is hypointense on T2-weighted sequences, because of melanin pigment dispersion.
- Panuveitis and endophthalmitis represent frequent inflammatory complications of proton beam radiotherapy.

## Introduction

This pictorial essay aims to provide the radiologists with awareness about diagnostic methods and therapeutic options of uveal melanoma. In the previous first instalment, we described ophthalmological and radiological imaging techniques to diagnose uveal melanomas with emphasis on the role of MR imaging and reviewed MR imaging findings of uveal melanoma.

In the present second instalment, we discuss the therapeutic management of uveal melanoma, describing the main ocular-conserving radiotherapic techniques and their indications. Finally, we present an overview of the effects of radiations on neoplastic tissue and of ocular complications following radiotherapy, using examples from our institution.

## Therapeutic management

The purposes of uveal melanoma treatment are to avoid the metastatic dissemination and to preserve the eye with useful vision [1]. Treatment should be personalized keeping into account many factors such as the location, size and extent of the lesion, the condition of the fellow eye, patient’s comorbidities, needs and desires. Nevertheless, the treatment choice is difficult due to the broad range of clinical settings and therapeutic chances, and the paucity of scientific evidence and guidelines.

Since different studies have demonstrated no significant difference in terms of survival rate between eye-preserving therapies and surgery, in recent years globe-sparing techniques are often preferred to surgical ones [2]. In this regard, it is important to emphasize that, because of continuous advances and technical improvements, the indications of radiation therapy (Table 1) are constantly subject to periodic update and modifications [3–12].

**Table 1:** Eye-preserving therapies of uveal melanoma

Technique	Method	Effects	Indication	Main complications	Limitations
*Laser therapy (LT)*					
Laser photocoagulation	Xenon or argon laser	Rise temperature inducting denaturization of proteins and cellular apoptosis	Small-sized tumours (< 3 mm)	Retinal vein occlusion, vitreous hemorrhage, optic atrophy, thrombotic glaucoma, macular involvement, cystoid macular oedema	No more used (superseded by TTT) because poor tissue penetration and multiple treatment sessions
Transpupillary thermotherapy (TTT)	Infrared diode laser (810 nm)	Rise temperature (45°–60°) inducting cytotoxic effects, vascular occlusion and tumour necrosis	Tumours thickness < 4 mm Distance from the fovea > 3 mm Not touching the optic disc Adjuvant treatment combined with other eye-sparing therapy	Retinal oedema Fine intraretinal hemorrhage	Amelanotic or poor melanotic choroidal melanoma Long-term possibility of recurrence and/or high metastatic risks Limited tissue penetration of 4 mm
*Radiotherapy (RT)*					
Brachytherapy (BRT)	Saucer-shaped plaque, sutured to the sclera, containing radioactive sources (62–70 Gy to the tumour apex): X-ray isotopes; γ-ray isotopes: *Cobalt-60, Palladium-103, Iridium-192, Iodine-125*; β-ray isotope: *Ruthenium-106*	Radiation-induced irreversible DNA damage that leads to cell death, cell cycle redistribution and microenvironment changes	AJCC-UICC T1, T2, T3 and T4a-d Basal diameter tumor < 16 mm Tumor thickness < 11 mm Supero-temporal quadrant	Radiation-induced retinopathy, cataract and maculopathy, secondary glaucoma, vitreous hemorrhage and retinal detachment, scleral necrosis, diplopia, strabismus, involvement of extra-ocular muscles	Large tumors (diameters > 15 mm; height > 10 mm) Distance from optic disc < 2 mm Limited tissue penetration of 4–5 mm Not eligible in case of: Blind painful eyes Extraocular extension (T4e) Limited light perception Damage to surrounding normal choroid
Charged-particles beam radiotherapy	Tumor irradiation with charged particles, protons and helium ions (53–70 Gy), over 4 consecutive days	*Bragg Peak*: particles release ionizing radiation when they stop traveling High dose of radiation leading to DNA damage and subsequent cell death	Basal diameter tumor < 28 mm Tumor thickness < 14 mm Neoadjuvant therapy before surgical resection	Retinopathy, rubeosis iridis, cataract, uveitis, optic neuropathy, maculopathy, dry eye, loss of eyelashes, retinal detachment, keratopathy	Low rates of visual prognosis and eye conservation for large melanomas Involvement of lacrimal glands (supero-temporal quadrant lesion)
*Stereotactic radiotherapy (SRT)*					
Gamma-Knife and Cyber-Knife	Stereotactic and robot-assisted radiosurgery with fractionated ionizing radiation (total dose of 30–50 Gy) delivered to a relatively small target, decreasing progressively at the margins (25–35 Gy)	Extremely focused dose of radiation leading to DNA damage and subsequent cell death	Tumor confined to the eye, sparing extra-ocular tissue (T4e) Juxtapapillary uveal melanoma Patients not eligible for BRT or surgery	Cataract, dry eye disease, vitreous hemorrhage, radiation retinopathy, radiation maculopathy, optic neuropathy, neovascular glaucoma	Lower availability Ocular fixation High rates of radiation-induced retinopathy and neovascular glaucoma
Linear accelerator (LINAC)	Stereotactic fractionated and hypofractionated photon radiotherapy (50–70 Gy in 5–7 Gy single-dose fractions)	Extremely focused dose of photon leading to DNA damage and subsequent cell death	Tumor confined to the eye, sparing extra-ocular tissue (T4e) Juxtapapillary uveal melanoma Patients not eligible for BRT or surgery	Conjunctivitis, skin reaction, maculopathy, cataract, ischemic retinopathy, glaucoma, retinal detachment, corneal ulcer, vitreous hemorrhage, optic nerve damage	Lower availability High rates of radiation-induced retinopathy and neovascular glaucoma

### Laser techniques

Laser methods induce necrosis into neoplastic tissues. However, because of their drawbacks represented by poor tissue penetration and requirement for multiple treatment sessions, laser techniques have very limited indications in the treatment of uveal melanomas [13].

#### Laser photocoagulation therapy

Previously used to treat small choroidal melanomas, currently laser photocoagulation has been largely replaced by transpupillary thermal therapy. Xenon arc photocoagulation showed a better tumor control than argon laser photocoagulation; however, the latter demonstrated a lower risk of complications (retinal traction, gliosis) [2, 13, 14].

#### Transpupillary thermal therapy (TTT)

In TTT, an infrared light (diode laser) is employed to target a lesion through the pupil. This laser technique is feasible for small- and medium-sized flat pigmented uveal melanomas of the extramacular, extrapapillary region and can be used alone or prior to plaque radiotherapy (sandwich therapy) [2, 13, 14]. TTT has the advantage of good visual prognosis, although a high rate of long-term recurrence (up to 29% at 5 years) must be kept into account [15].

### Radiation therapy

Radiation therapy is the most widely used globe-conserving therapy for uveal melanoma and encompasses brachytherapy (episcleral plaque radiotherapy) with various radioactive sources, charged-particle beam therapy and stereotactic radiosurgery. The different kinds of radiotherapy differ not only on the basis of the type of radiation employed, but also with respect to the ocular morbidity they cause [4].

#### Episcleral plaque radiotherapy

Currently, episcleral plaque radiotherapy represents the most commonly used kind of eye-preserving therapy for uveal melanoma [3]. It is a sort of brachytherapy that employs different radioisotopes:

- gamma-ray emitting isotopes: iodine-125 (^125^I), palladium-103 (^103^Pd), iridium-192 (^192^Ir) and cobalt-60 (^60^C0);
- beta-particle-emitting isotopes: ruthenium-106 (^106^Ru).

A custom-designed curvilinear radioactive plaque is provisionally sutured onto the sclera, in correspondence of the lesion, in order to administer trans-scleral radiation to the tumor (dose of 62–70 Gy). After a temporal interval of 2–7 days (variable according to the applied radioisotope), the radioactive device is removed [2, 3, 13, 14, 16–18]. According to the Collaborative Ocular Melanoma Study (COMS), the plaque should outstrip the tumor margins by 2 mm [19]; however, Nag et al. described a custom-designed plaque able to shorten the margin of disease-free tissue around the lesion up to 1 mm, thus decreasing the area of irradiated healthy retina [20]. Brachytherapy allows to reach tumor control and eye salvage in about 98% and 95% of cases, respectively [14]. Moreover, recent advances in plaque design allow to treat large uveal melanomas and melanomas surrounding or even encompassing the optic nerve [3]. Recently, 3D printing methods have further improved the capability of plaque design [21].

#### Charged-particle beam radiotherapy

In this kind of teletherapy, an extremely collimated beam of protons or helium ions is used to concentrate a high and homogenous dose of radiation to the lesion; because of Bragg peak effect, the density of ionization of protons is concentrated in correspondence of the end of their path and the radiation dissipates beyond the tumor boundaries, thus reducing injuries to adjacent tissues. Adjacent tissue sparing is particularly efficient at the sides and posteriorly to the lesion, whereas at the beam entrance the dose of radiation is relatively high; therefore, tissues located along the beams route are exposed to a considerable radiation dose too [2, 13, 14, 22, 23].

Proton-beam radiotherapy was employed for the first time in the treatment of uveal melanoma in 1975 [24]. Currently it is used as primary treatment for melanomas of various size and location, as neoadjuvant therapy before surgical resection and as an effective technique to treat recurrent uveal melanomas previously treated with other forms of radiotherapy or surgery. In the treatment of uveal melanoma, proton-beam radiotherapy has a wider range of indications than other forms of radiotherapy; nevertheless, it is available only at a few centers worldwide [22, 25–28].

**Fig. 1:**
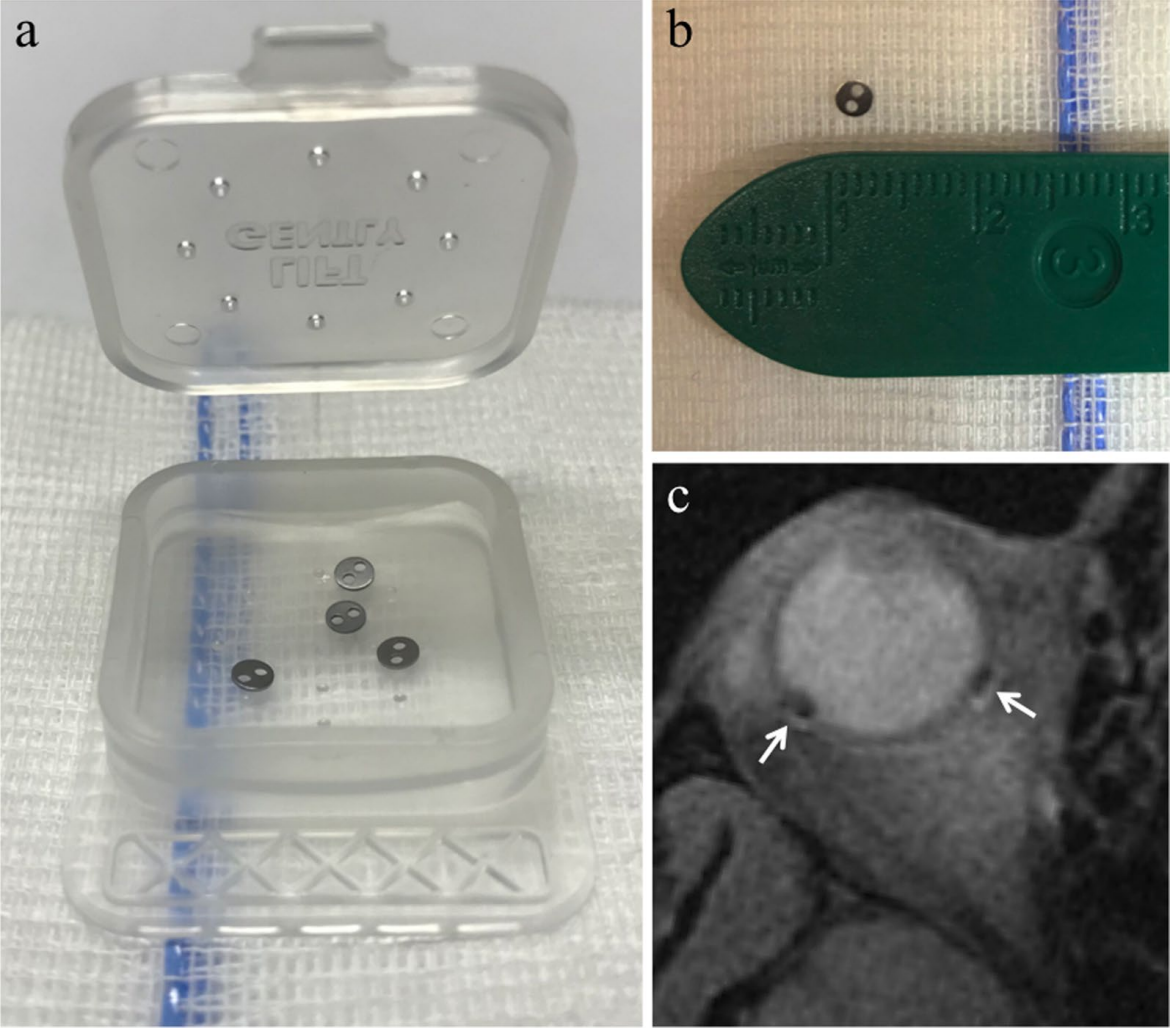
Tantalum clips for proton-beam radiotherapy. **a, b** Tantalum clips (2.5 mm in diameter). **c** Fat-suppressed T1-weighted image shows the artefact produced by the clips (white arrows); the artefact is negligible since tantalum is a nonmagnetic metal

**Fig. 2.**
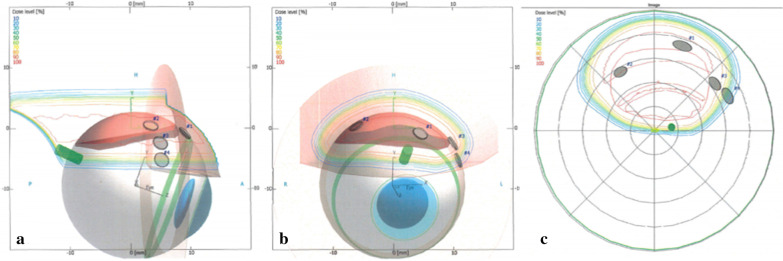
3D computer model of the patient’s eye/tumor for proton-beam radiotherapy. Eyeplan treatment planning system: (**a**) eye side, (**b**) beam axis and (**c**) optical-axis-centered fundus isodose views

Proton beam therapy protocol includes the following phases:

1. For tumor involving the choroid and/or ciliary body, three-to-five tantalum clips (2.5 mm in diameter) are sutured to the sclera to delimit the tumor borders (Fig. 1); iris melanomas don’t need the placement of tantalum markers due to their visually assessable extension.
2. On the basis of ocular biometry, ultrasound findings, color photography, cranial X-rays and intra-operative marker measurements, a 3D computer model of the patient’s eye/tumor is generated (also showing the proton beam, eyelids and optic nerve) in order to plan an individualized irradiation field. This planning needs a close cooperation among the ophthalmologist, the radiation oncologist and the medical physicist (Fig. 2) and is mandatory for local tumor control.
3. A total radiation dose of 53–70 Gy is delivered in four consecutive daily fractions (30 s each). During the treatment, patient’s head immobilization is ensured by means of a custom-made face mask and dental bite block. Patient’s eye positioning is maintained constant through a visual target; irradiation is automatically temporarily suspended whenever the patient involuntarily moves the affected eye from the planned position. Safety margins may range from < 2 mm (for juxtapapillary–juxtafoveal lesions) to 4 mm (for ciliary body melanomas) [22, 23, 25, 29].

Charged particle radiotherapy allows to reach tumor control in about 95–98% of cases [14]. In the event of local recurrence after proton beam therapy, a second treatment with proton beam is still possible in very selected cases; nevertheless, a second radiotherapy entails a risk of complications far higher than the initial one [23]. Ocular inflammation represents one of the main complications of proton beam therapy, being encountered in about 28% of patients [30].

#### Differences between proton beam radiotherapy and plaque brachytherapy

A series of differences between proton beam radiotherapy and plaque brachytherapy must be kept into account when choosing the most suitable ocular-conserving radiotherapic treatment.

Owing to the physical properties of protons, proton beam radiotherapy has theoretical advantages as compared with other kinds of radiation therapy:

- highly collimated beams with very low scattering (relative sparing of healthy tissues adjacent to the beam path),
- boosted radiation dose at the end of the pathway,
- optimal and uniform radiation dose delivery at the level of the tumor,
- healthy tissue relative sparing distally and, to a lesser extent, proximally to the targeted lesion [25, 31, 32].

All these features of accelerated proton beam are specifically relevant in the treatment of uveal melanoma, since this malignant neoplasm is relatively radioresistant and its radiotherapy requires high radiation dose [23].

When treating tumors with noticeable size, challenging shape and location, proton beam radiotherapy may be more feasible than plaque brachytherapy since the former lessens the risk of recurrence and injury to the optic disc and fovea [25].

Plaque brachytherapy is suitable for small- and medium-sized uveal melanomas because the radiation dose decreases with increasing distance from the radioactive plaque; for this reason, in large lesions the tumor apex does not receive a sufficient radiation dose, due to the excessive distance from the radiation source. Lesions close to the optic nerve head are challenging to treat with brachytherapy as well; for such lesions proton beam therapy represents the best treatment option [16, 33].

Tantalum markers’ positioning for proton beam therapy needs lower surgical precision than insertion of plaque for episcleral brachytherapy [25]. When facing iris melanomas, proton beam radiotherapy shows more favorable dosimetric profile than brachytherapy, besides does not require any surgical procedure inasmuch iris lesions do not require tantalum markers placement [25]. Moreover, conversely from brachytherapy, proton beam radiotherapy does not imply handling of radioactive material by ophthalmologists [22].

Tumor regression is quicker after plaque brachytherapy than after proton beam radiotherapy, presumably as a consequence of a higher radiation dose provided to the tumor base through plaque radiotherapy [4].

Usually complications affecting the anterior segment are more common following external beam radiotherapy then after plaque brachytherapy [34]. According to Tseng et al., patients undergoing proton beam therapy would have higher rates of vision loss, neovascular glaucoma and enucleation as compared with patients treated with plaque brachytherapy [35].

Secondary enucleation rate at 5 years after radiotherapy is higher with proton beam therapy (5.4–14%) than with plaque brachytherapy (4%) [16, 25, 36].

Local tumor recurrence rates at 5 years range from 2% to 8.4% following proton beam therapy [29] and from 6 to 13% following plaque brachytherapy [14].

Proton beam therapy is more expensive as compared with plaque brachytherapy, with a treatment cost per patient of about €30,000 and €6,000, respectively. Furthermore, proton beam therapy is still less available than brachytherapy [16].

#### Stereotactic radiosurgery

Stereotactic radiotherapy involves the use of multiple photon beams focused towards the tumor from different directions [14]. Tumor location and borders are detected before the treatment by means of cross-sectional imaging (CT and MRI). Stereotactic radiotherapy has some advantages compared to the other forms of radiotherapy, such as no need for preoperative surgical marking and cost-effectiveness. On the other hand, however, proton beam therapy allows to better spare surrounding healthy tissues from radiation injuries [2].

Stereotactic radiotherapy encompasses different modalities of irradiation such as Gamma Knife, Cyber Knife (both first used to treat brain tumors) and linear accelerator.

Although *Gamma Knife* has demonstrated successful in treating uveal melanomas unsuitable for plaque brachytherapy, it is not routinely used because of its high rates of radiation-induced inflammation, radiation retinopathy and neovascular glaucoma [2].

In uveal melanoma patients in which neither other forms of radiotherapy nor local resection are suitable, *Cyber Knife* allows to reach local tumor control in about 74% of cases [2].

### Surgery

#### Local resection

Local resection implies the surgical excision of the tumor and allows to spare the globe and vision; moreover, it offers histopathologic confirmation and cytogenetic analysis for prognostic purposes. This technique may be pursued for circumscribed choroidal melanomas not suitable for radiotherapy because of juxtapapillary location or large size and can be performed through a trans-retinal (endoresection) or trans-scleral (exoresection) approach. However, the main indication of local resection is represented by iris and ciliary body melanomas; in particular, iridectomy is indicated for iris melanomas not involving the angle, whereas iridocyclectomy is indicated for lesions extending to the angle and ciliary body. Rhegmatogenous retinal detachment, vitreous hemorrhage and tumor recurrence represent major complications of local resection [2, 14].

Neoadjuvant radiotherapy is performed in some centers in order to reduce enucleation-induced tumor seeding [13]. Another approach in the treatment of uveal melanoma is endoresection in combination with plaque brachytherapy [3, 37].

#### Enucleation

**Fig. 3:**
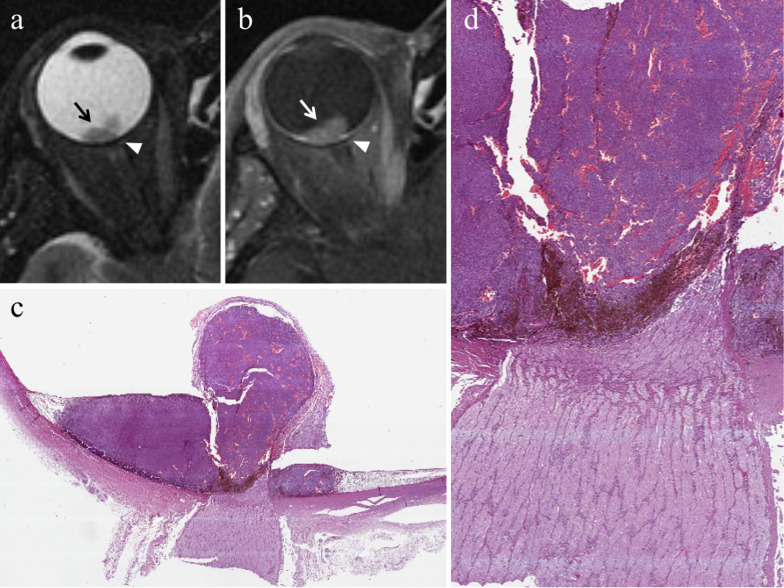
A 55-year-old woman with a choroidal melanoma of the right eye infiltrating the optic disc. Axial (**a**) T2-weighted turbo spin-echo STIR and (**b**) contrast-enhanced fat-suppressed T1-weighted images demonstrate an intraocular lesion along the posterior aspect of globe (white arrows), infiltrating the optic disc (white arrowheads). **c** On low magnification, histological examination shows a strong overlap with MR imaging: a poorly pigmented mass located in the posterior segment of the eye, at the level of the optic disc (H&E, original magnification 25×)**. d** Higher magnification confirms the MR findings, demonstrating an early infiltration of the emergence of the optic nerve by the melanoma (H&E, original magnification 50×)

The ordinary treatment until 1960, enucleation is currently restricted to advanced uveal melanomas (basal diameter > 20 mm, thickness > 12 mm), blind and painful eyes because of tumor complications, and uveal melanomas with orbital involvement or optic nerve invasion (Fig. 3). In this latter case, the procedure is performed together with the resection of a long portion of optic nerve [2, 13, 14, 38].

*Enucleation* can be *primary*, in patients who do not underwent any other kind of treatment, or *secondary* in patients previously treated with eye-preventing treatments (plaque brachytherapy or charged-particle beam radiotherapy). Reasons for secondary enucleation can be represented by treatment-related complications (neovascular glaucoma, poor vision, pain, chronic inflammatory processes) or treatment failure for local control (tumor progression, extrascleral extension, local recurrence) [22, 39, 40].

#### Orbital exenteration

Orbital exenteration is a surgical procedure that implies excision of the globe, extraocular muscles, nerves and periocular adipose tissue. At present, orbital exenteration is justified for uveal melanomas with massive orbital extension, associated with a blind and painful eye [13, 14].

## Effects of radiotherapy on uveal melanomas

Overall, ionizing radiation may damage cells both directly, by breaking molecular bonds, and indirectly, through the development of toxic-free radicals.

Cell death can be determined through the following mechanisms:

- increased membrane permeability (radiation dose ≤ 30 Gy);
- breaking of cellular membrane with inflow of extracellular fluid into the cell (radiation dose > 30 Gy);
- disruption of cytoplasmic lysosomes with spillage of enzymes that digest cellular structures (radiation dose 5–100 Gy);
- disruption of mitochondria and interruption of adenosine triphosphate (ATP) production (radiation dose 5–100 Gy) [4].

Apoptosis represents another process of cell death and takes place when DNA alteration triggers the gene TP53 [4].

### Effects of radiotherapy on neoplastic tissue: pathologic features of irradiated uveal melanomas

Basically, tumor regression relies on two factors:

- the type of radiation therapy,
- the tumor cell-doubling time (shorter in high-grade tumors) [4].

As for the effect of radiations on neoplastic cell viability, radiotherapy may theoretically determine: (1) necrosis of the tumor tissue with subsequent reabsorption of necrotic cellular debris by macrophages; (2) sterilization of the tumor, namely suspension of mitotic activity, growth interruption and impairment of its metastasizing capability [31, 32].

Thanks to the description, reported by some authors [31, 32, 41, 42], of the histological and ultrastructural alterations observed in enucleated eyes after prior proton beam therapy, it is possible to understand the effects of radiotherapy on both tumor tissue and healthy ocular tissues.

Uveal melanoma regression after radiotherapy is a multifactorial event encompassing multiple components. In particular, the mechanism by which proton beam therapy produces its effect on tumor tissue lies on three main aspects: (1) direct cytotoxic action of the radiations on neoplastic cells; (2) indirect effect through impairment of neoplastic vascular supply resulting in ischemia and consequent progressive cell death; (3) immunologic response boosted by radiation-damaged tumor tissue [32, 41]. Previous authors observed a direct relationship between the length of the interval from irradiation to enucleation and the degree of resulting histologic alterations [32, 41].

#### Direct radiation-related cytotoxic effect: necrosis—immune response—fibrosis

**Fig. 4:**
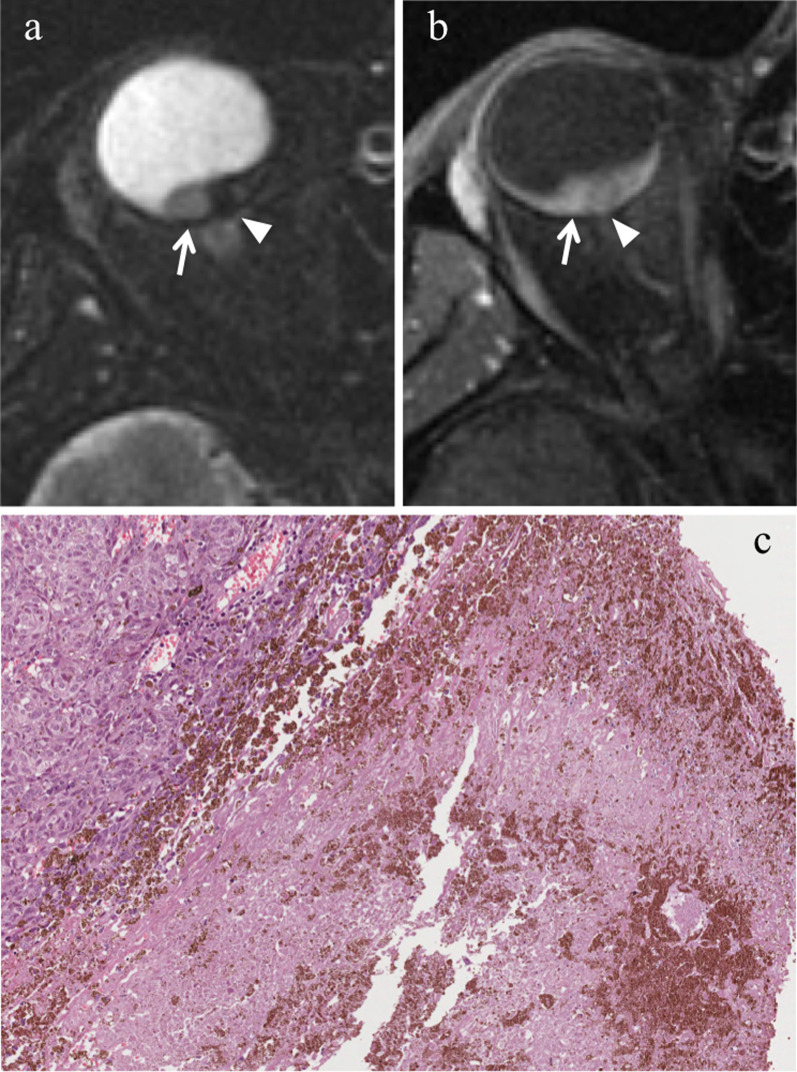
A 29-year-old man with a choroidal melanoma of the right eye treated with proton-beam radiotherapy. The patient underwent secondary enucleation about three years after radiotherapy. Axial **a** T2-weighted turbo spin-echo STIR and **b** contrast-enhanced fat-suppressed T1-weighted images display an intraocular lesion along the posterior aspect of globe (white arrows). On T2-weighted image, a central well-marginated hypointense area is detectable within the mass (white arrowhead); it represents radiotherapy-related necrosis and its low signal intensity is due to the dispersion of melanin pigment. On contrast-enhanced T1-weighted image, the above-mentioned area appears relatively hypointense (white arrowhead) compared to the surrounding enhancing viable neoplastic tissue. **c** Histological examination showing an “abrupt transition” between a radiotherapy-related necrotic area with dispersion of melanin pigment (on the right) and the vital tumor tissue (on the left) (H&E, original magnification 100×)

**Fig. 5:**
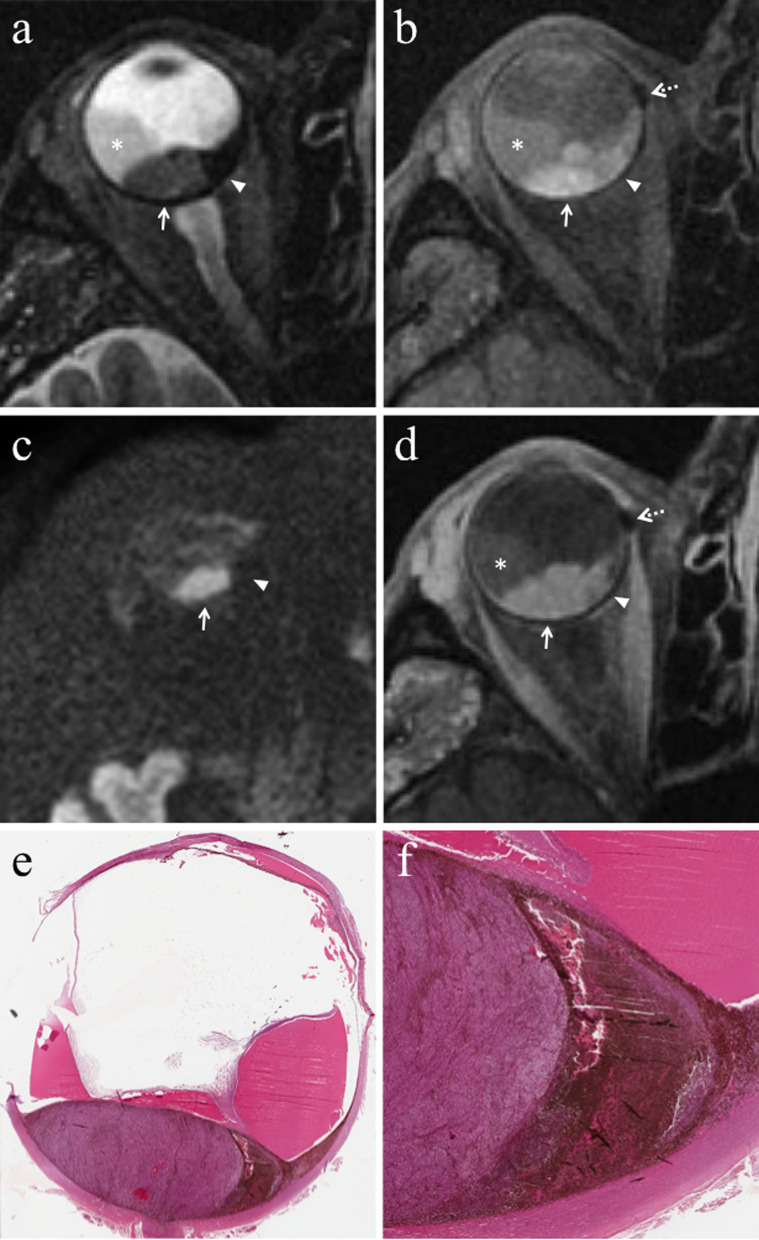
A 60-year-old man with choroidal melanoma of the right eye treated with proton-beam radiotherapy. The patient underwent secondary enucleation about three years after radiotherapy because of local recurrence. Axial **a** T2-weighted turbo spin-echo STIR, **b** fat-suppressed T1-weighted, (**c**) DW (b = 1000 s/mm^2^) and (**d**) contrast-enhanced fat-suppressed T1-weighted images show an intraocular mass along the posterior aspect of globe, at the level of the optic disc. The central and lateral portions of the lesion (white arrows) exhibit intermediate signal intensity on T2-weighted image and high signal intensity on T1-weighted image and represent viable tumor. The medial portion of the mass demonstrates low signal intensity on T2-weighted image and moderately high signal intensity on T1-weighted image; it represents radiation-induced necrosis with dispersion of melanin pigment, responsible for the low T2 signal. Note the well-defined border between the two distinct portions of the lesion, particularly evident on T2-weighted image. On (**d**) axial contrast-enhanced fat-suppressed T1-weighted image, the viable tumor demonstrates mild enhancement (white arrow) compared to relatively lower signal intensity of the medial necrotic part (white arrowhead). On **c** DW image, the viable neoplastic tissue displays high signal intensity (white arrow), a finding consistent with restricted diffusion due to high cellularity, whereas the necrotic part (white arrowhead) is hypointense, lacking of restricted diffusion. Laterally to the lesion a retinal detachment is detectable (white asterisks), with intermediate signal intensity on T2- and T1-weighted images, without enhancement after contrast agent administration. Along the medial outer edge of the sclera, a small metal artefact due to tantalum clip is appreciable (white dotted arrows in **b** and **d**). **e** Histological examination: low magnification showing a poorly pigmented melanoma, protruding into the posterior ocular segment and containing a necrotic component (on the right) (H&E, original magnification 25×); **f** Higher magnification demonstrating the "abrupt transition" between vital tumor tissue (on the left) and necrosis with abundant dispersed melanin (on the right) (H&E, original magnification 50×)

The distribution of necrotic areas within the tumor may range from no necrosis, to minimum proof of necrosis, to few, sparse foci of necrosis, to random, patchy or several diffused areas of necrosis, up to extensive necrosis. Necrotic areas of irradiated melanomas are characterized by pigment dispersion with storage of pigment-laden macrophages. This dispersion of melanin pigment is responsible for the MR appearance of radiotherapy-related necrosis that demonstrates a characteristic low signal intensity on T2-weighted sequences (Figs. 4 and 5) [43]. Necrotic areas are surrounded by chronic inflammatory cell infiltrates; these latter consist primarily of lymphocytes and, to a lesser extent, of plasma B cells and are thought to represent the result of immunological response against the neoplastic necrotic products [31, 32, 41].

**Fig. 6:**
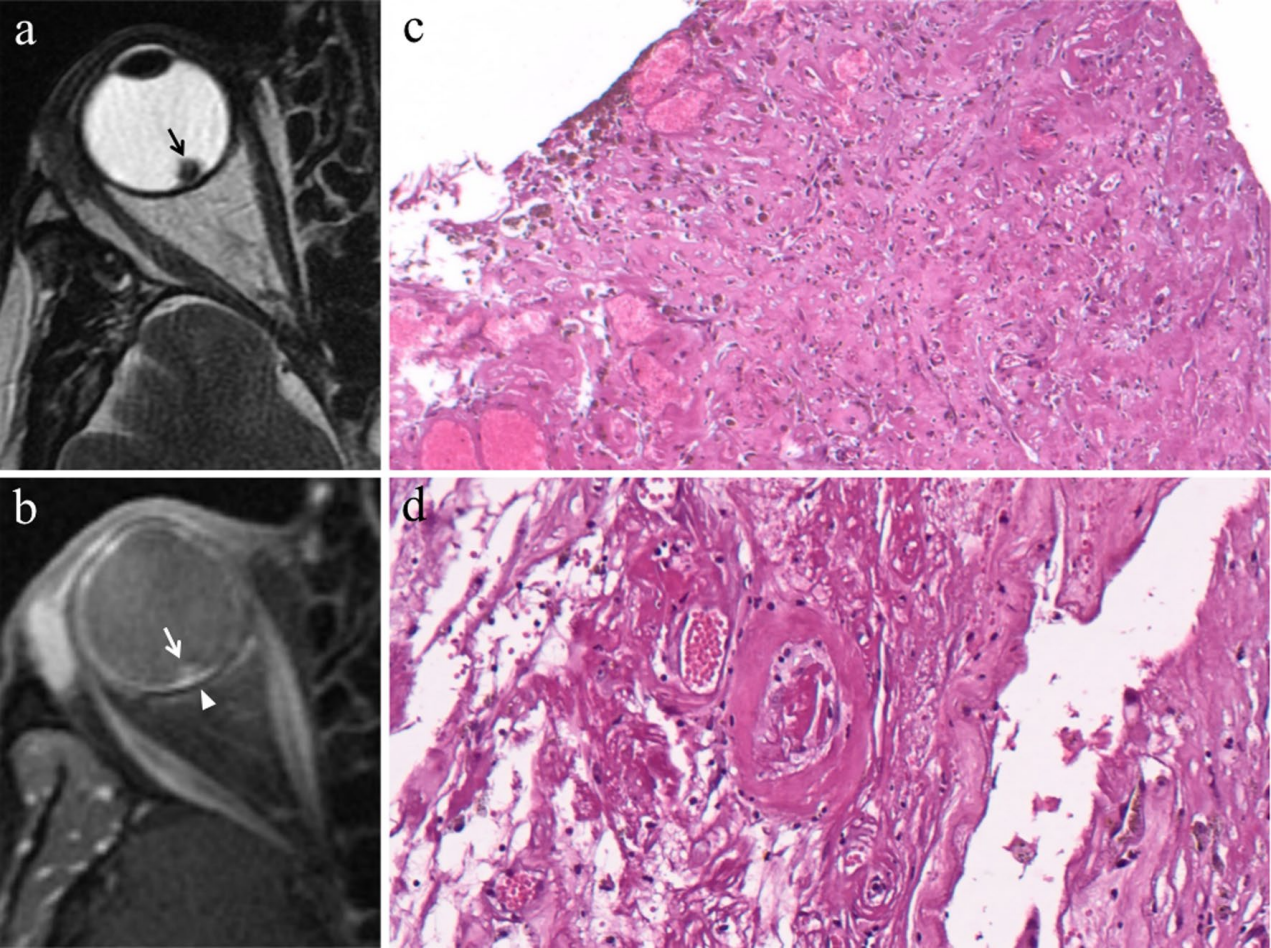
A 58-year-old man with residual fibrotic scar after proton-beam radiotherapy for choroidal melanoma of the right eye. The patient underwent secondary enucleation two years after radiotherapy because of neovascular glaucoma. **a** Axial T2-weighted turbo spin-echo STIR image demonstrates an intraocular small rounded hypointense mass along the posterior aspect of globe (black arrow). On **b** contrast-enhanced fat-suppressed T1-weighted image, the mass exhibits mild enhancement (white arrow). Note the pronounced enhancement of the choroid below the irradiated lesion due to radiation-related neoangiogenesis (white arrowhead). **c** Histological examination showing a well-circumscribed sclero-hemorrhagic nodule composed of ectatic vessels, fibrosis with scattered reactive fibroblasts and abundant component of melanophages; no neoplastic cells are visible (H&E, original magnification 200×). **d** On higher magnification, within the choroid below the abovementioned nodule, ectatic and thick-walled vessels are evident (H&E, original magnification 400×)

**Fig. 7:**
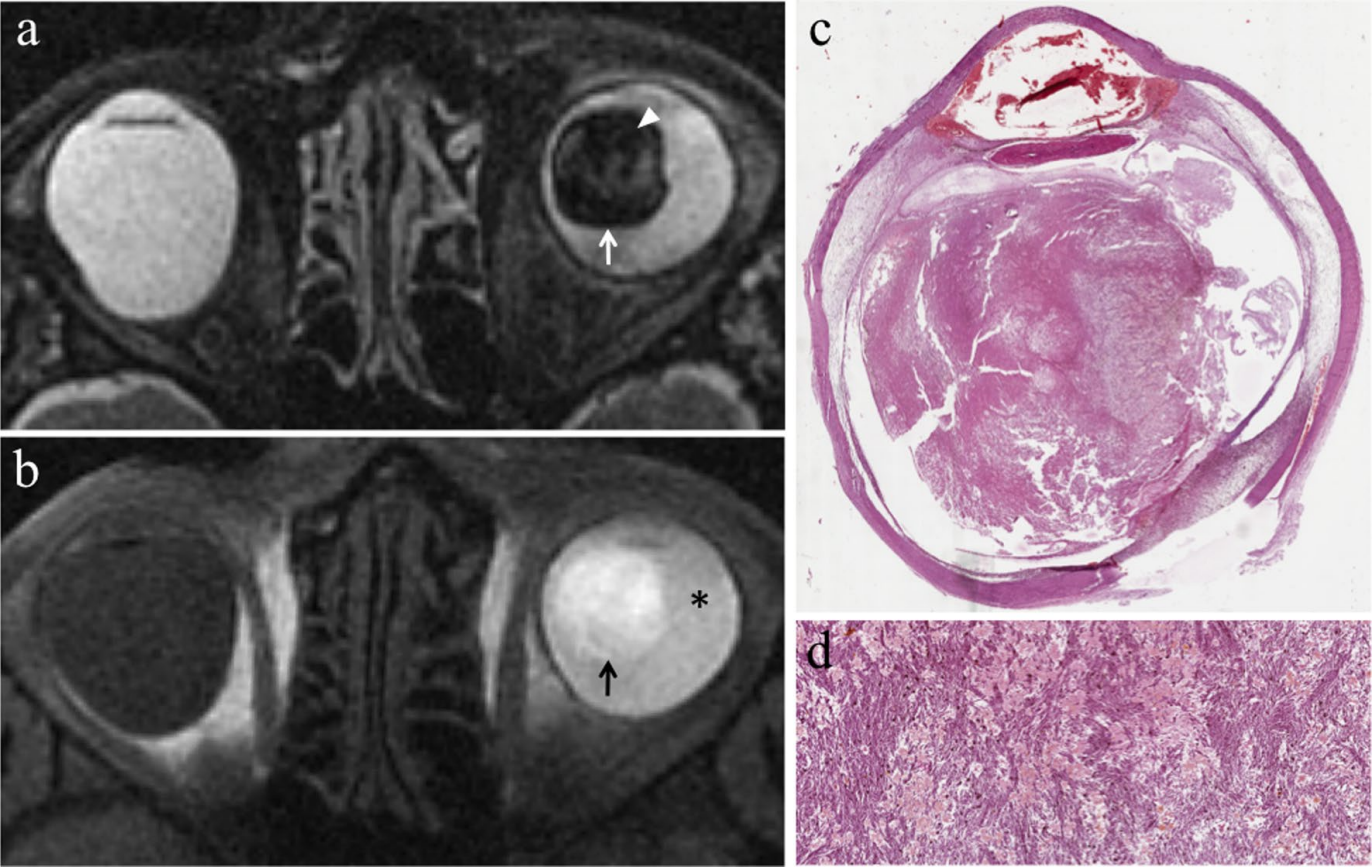
An 82-year-old man with a choroidal melanoma of the left eye treated with plaque brachytherapy. The patient underwent secondary enucleation seven years after radiotherapy because of painful eye and local recurrence. Axial **a** T2-weighted turbo spin-echo STIR and **b** fat-suppressed T1-weighted images. Along the medial aspect of the left globe an intraocular mass (arrows) exhibits inhomogeneous signal intensity with irregularly edged hypointense areas on T2-weighted image (white arrowhead in** a**), due to radiation-induced fibrotic alterations. The anterior chamber and the vitreous body of the left eye demonstrate high signal intensity on T1-weighted image (black asterisk in** b**), a finding consistent with extensive vitreous hemorrhage. Note the difference with the physiological water-like signal intensity of the contralateral eye. **c** Histological low magnification showing the heterogeneous appearance of the tumor, composed of poorly pigmented spindle cells, intermingled with dense and eosinophilic intratumoral fibrotic areas (H&E, original magnification 25×). **d** On higher magnification, the fibrotic nature of the above-described eosinophilic areas is well documented: neoplastic spindle cells are mixed to multiple deposits of dense collagen fibres, representing the main effect of radiotherapy (H&E, original magnification 100×)

Inflammatory changes occur early after irradiation, and their prevalence tends to decrease over time; on the other hand, fibrotic alterations (stromal collagen deposition), representing sequelae of inflammatory process, are a late-growing finding whose prevalence increases with time. At MRI, fibrotic changes demonstrate low signal intensity on T2-weighted sequences due to stromal collagen deposition (Figs. 6 and 7) [44].

The effect of host response to radiation is highlighted by the finding of inflammatory and fibrotic alterations that are more evident in irradiated tumors of patients undergoing secondary enucleation after failed radiotherapy than in patients with primary enucleation. The observation that remaining viable tumor of irradiated lesions shows less mitotic activity as compared to melanomas undergoing primary enucleation reflects the result of radiation-related direct cytotoxicity [39]. In particular, in the context of irradiated uveal melanomas, mitoses gradually decrease and would no longer be recognizable more than 30 months since irradiation [44].

However, it is important to remember that necrosis may also represent a spontaneous biological event occurring in nonradiated melanomas, especially large-sized tumors [32].

#### Vascular changes

Tumor blood vessel damage occurs relatively earlier after irradiation and tends to remain constant over time [44]. Endothelial cells of the tumor capillaries appear swollen, ruptured, with narrowing of the lumen; the basement membrane is thickened because of collagen deposition. Fibrin accumulation into the perivascular interstitial space coexists. The lumen of some tumor vessels is occluded by fibrin deposition and hyalinized material. Collapse of "sinusoidal" vessels with leakage of red blood cells is often observed along with vascular thrombi. Several vessels are surrounded by pigment-laden macrophages and chronic inflammatory cells [31, 32, 41, 42]. These alterations determine vascular occlusion and consequently tumor ischemia.

#### Electron microscopic findings

Ultrastructural alterations suggesting radiation-induced cellular damage may be detectable at electron microscopy, even in the absence of light microscopic findings of tumor regression.

These ultrastructural changes include:

- degeneration and reduction in number of mitochondria;
- hardly recognizable Golgi’s apparatus;
- patchy melanin granules;
- augmented cytoplasmic filaments;
- storage of lipid vacuoles in cytoplasm;
- presence of phagolysosomes and autophagic vacuoles within cytoplasm;
- cell nuclei appear pyknotic, hyperconvoluted, fragmented, amitotic, with invagination and cleavage of nuclear membrane [31, 41].

Both necrotic and ultrastructural alterations are more evident in a pericapillary location and in close proximity to vascular impairment, indicating that radiation-induced damage on melanoma vasculature may have a pivotal role in tumor regression [31, 41].

In brief, as compared with tumors undergoing primary enucleation, irradiated uveal melanomas demonstrate more necrotic, inflammatory, fibrotic alterations and blood vessel damage as well as less mitotic figures [44].

### Evolution of the tumor following radiotherapy

Although melanoma regression after proton beam therapy is somewhat slow with lesions decreasing in size within 2 years, usually regression takes place mainly during the first 12 months [23, 30, 45–47]. After irradiation, responding uveal melanomas regresses in the form of a residual inactive scar of various sizes [23]. Initially, after the radiation therapy, a temporary growth of tumor dimension may occur, as a consequence of an interstitial edema. However, subsequently, phenomena related to ischemic necrosis outweigh, leading to tumor shrinkage; eventually, fibrosis develops [4, 23]. According to some authors [48], highly aggressive lesions demonstrate an early reduction in tumor volume.

Local tumor control is attested by the lack of tumor growth during follow-up [23].

The majority of the recurrences take place during the 3 years after radiotherapy in the form of three different patterns:

- recurrence at the margins of the lesion, the most frequent, presumably related to insufficient dose of radiation at the boundary of radiation field;
- recurrence at the inferior periphery, far from the initial location of the lesion, because of migration of neoplastic cells inside retinal detachment;
- recurrence into the radiation field, because of tumor radioresistance and accounting for late recurrences over 5 years from radiotherapy [23, 29].

## Effects of radiotherapy on ocular and periocular tissues

The main predictors of ocular complications after plaque brachytherapy and proton beam therapy are: tumor thickness, distance between the tumor and optic nerve, and radiation dose [35]. MR appearance of radiotherapy-related ocular and periocular complications is described in Table 2.

**Table 2:**
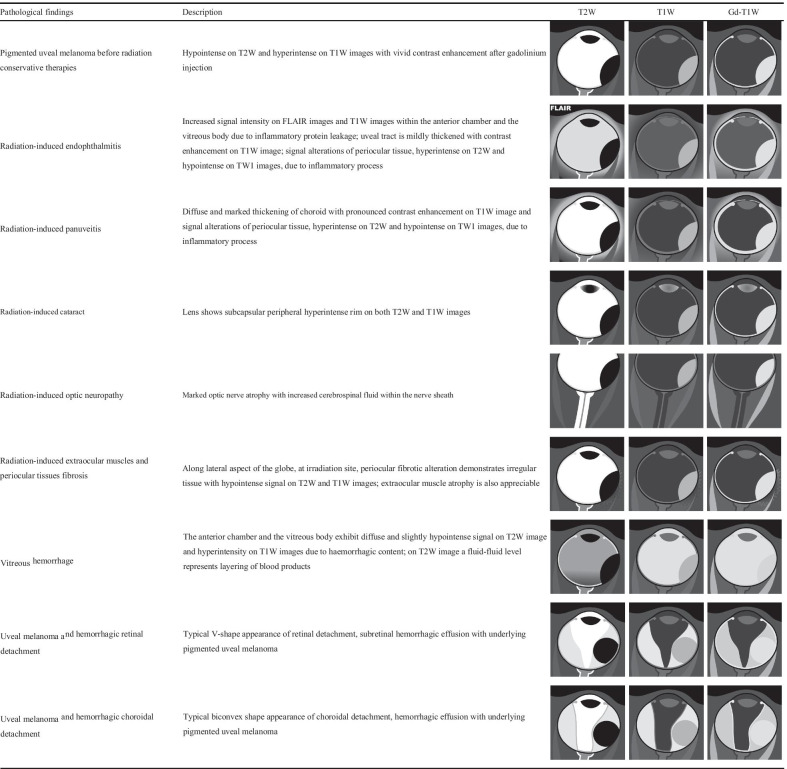
Chart summarizing MR imaging features of radiotherapy-related ocular and periocular complications of uveal melanoma

### Radiation-related intraocular inflammation

Intraocular inflammation represents a not uncommon finding after proton beam radiotherapy being observed in 28% of irradiated eyes up to 5 years after treatment [30]. The clinical features usually comprise mild anterior uveitis with cells and flare, increase of intraocular pressure and, more seldom, mild anterior vitreous inflammation. The clinical course is usually favorable to the point where inflammation is often diagnosed at the stage of sequelae, represented by pigmented keratic precipitates and posterior synechiae [30]. Nevertheless, chronic inflammation may determine nonreversible vision loss because of photoreceptor impairment and retinal atrophy [40].

Risk factors associated with the development of intraocular inflammation are tumor-related: location anterior to the equator or involving the equator, initial tumor height > 5 mm, diameter > 12 mm, tumor volume > 0.4 cm^3^ [30].

The pathogenesis of the inflammation following radiotherapy is not entirely known and seems to be related to various factors: direct effect of radiations on the ciliary body, irradiation of a large volume of the eye, breakdown of the blood-aqueous and blood-retinal barriers due to vascular damage, tumor necrosis [30, 40, 49]. In particular, the relationship between tumor necrosis and intraocular inflammation would be supported by the fact that uveal melanoma regression after proton beam therapy occurs after the first 12 months and inflammatory alterations are more evident during the same lag time [30, 46, 47].

Early treatment of inflammatory processes following radiotherapy is mandatory to prevent neovascular complications (neovascular glaucoma and retinopathy) and to preserve vision [40].

**Fig. 8:**
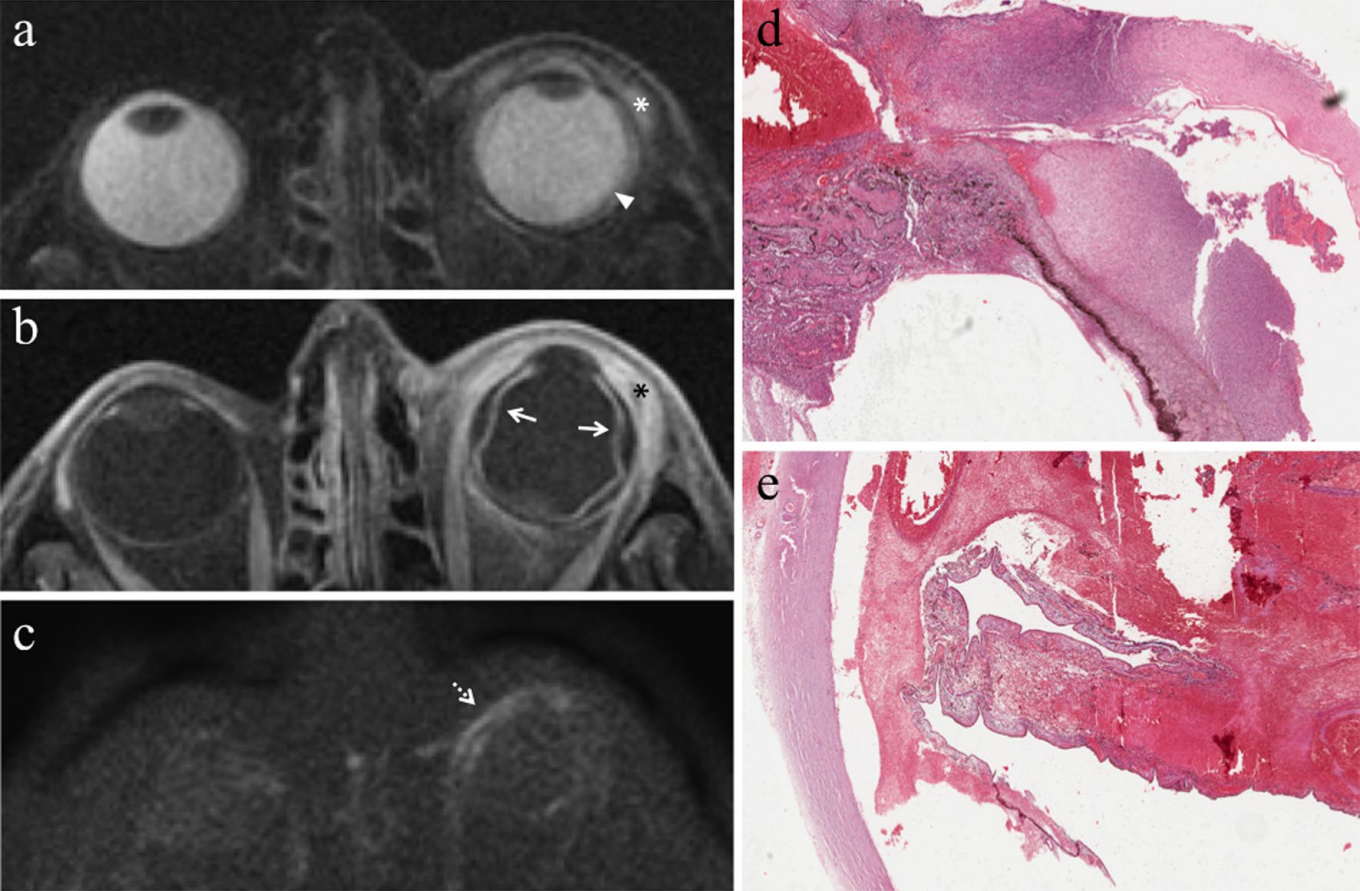
A 72-year-old woman with radiation-induced panuveitis and diffuse choroidal detachment after proton-beam radiotherapy for uveal melanoma of the left eye. The patient underwent secondary enucleation four years after radiotherapic treatment because of radiation-related inflammatory complications. Axial (**a**) T2-weighted turbo spin-echo STIR and **b** contrast-enhanced fat-suppressed T1-weighted images. The choroid is diffusely thickened and detached and displays pronounced enhancement after contrast agent administration (white arrows in** b**). On T2-weighted image, an exudative collection into the suprachoroidal space is detectable along the lateral aspect of the left eye (white arrowhead). Note the diffuse edematous thickening of the periocular tissues that appear hyperintense on T2-weighted image (white asterisk) and demonstrate marked enhancement on contrast-enhanced T1-weighted image (black asterisk). On **c** axial DW image (b = 1000 s/mm^2^), the periocular tissues and detached choroid exhibit restricted diffusion with high signal intensity (white dotted arrow). **d** Medium magnification showing the histological equivalent of radiologically identified panuveitis: the suprachoroidal compartment is entirely replaced by an acute/suppurative inflammatory process, rich in neutrophils (H&E, original magnification 50×). **e** Histopathology confirms the presence of a radiotherapy-related choroidal detachment (H&E, original magnification 50×)

Uveitis may involve the iris (iritis), the ciliary body (cyclitis) or the choroid (choroiditis); however, it often affects more than one or all three of the aforementioned structures (panuveitis) and extends to the retina and sclera as well. In case of panuveitis, MRI shows diffuse thickening of both the anterior segment and the posterior wall of the globe that demonstrate pronounced enhancement on contrast-enhanced fat-suppressed T1-weighted images (Table 2, Fig. 8) [50].

**Fig. 9:**
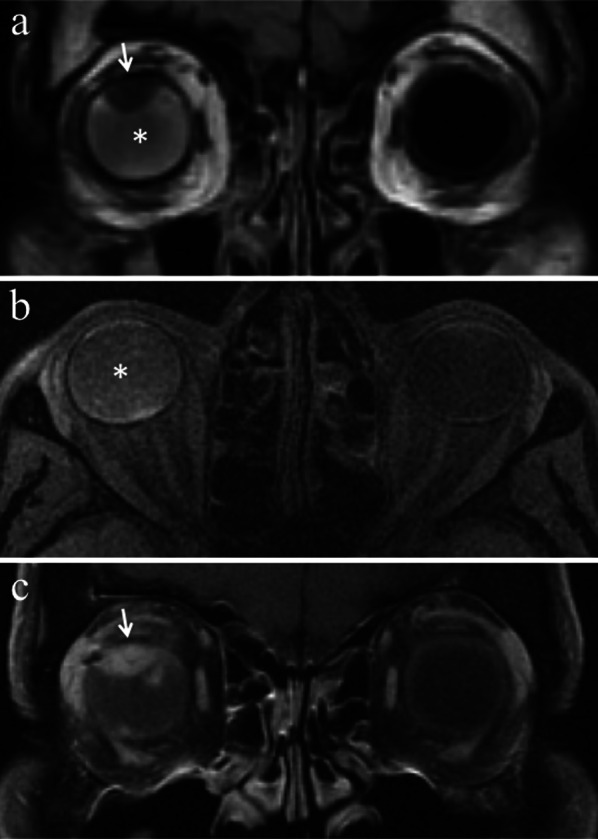
A 37-year-old man with radiation-induced endophthalmitis after proton-beam radiotherapy for choroidal melanoma of the right eye. The patient underwent secondary enucleation three years after radiotherapy because of drug-resistant neovascular glaucoma and painful eye, associated with local recurrence. **a** Coronal T2-weighted FLAIR (from brain MRI scan) and **b** axial fat-suppressed T1-weighted images show increased signal intensity of the vitreous body of the right eye (white asterisks) due to vitreous inflammation with protein leakage; note the difference with the physiological water-like signal intensity of the contralateral eye. Along the superior aspect of the right globe, a dome-shaped intraocular mass is detectable, consistent with local recurrence of the choroidal melanoma (white arrow in **a**). On (**c**) coronal contrast-enhanced fat-suppressed T1-weighted image, the mass demonstrates enhancement (white arrow)

Endophthalmitis concerns to an intraocular inflammatory process affecting the anterior chamber and the vitreous body. At MR, the vitreous body demonstrates increased signal intensity on T2-weighted fluid-attenuated inversion recovery (FLAIR) images and precontrast T1-weighted images, due to protein leakage from retinal and choroidal vessels into the vitreous coupled with vitreous inflammation (Table 2, Fig. 9). The whole uveal tract can be thickened as well, displaying noticeable enhancement on contrast-enhanced fat-suppressed T1-weighted sequences. Both panuveitis and endophthalmitis can be accompanied by retinal and choroidal detachment (Fig. 8) [50].

### Sclera and episclera

Chronic inflammatory scleral and episcleral deposits, mainly composed of pigmented migrating macrophages and their debris, have been described close to the tumor in most patients following radiotherapy [34, 41]. Scleral necrosis represents an unusual complication of both plaque brachytherapy and proton beam radiotherapy, its severity ranging from scleral translucency to perforation. Tumor thickness > 6 mm and ciliary body invasion are considered risk factors for scleral necrosis [4].

### Cornea

**Fig. 10:**
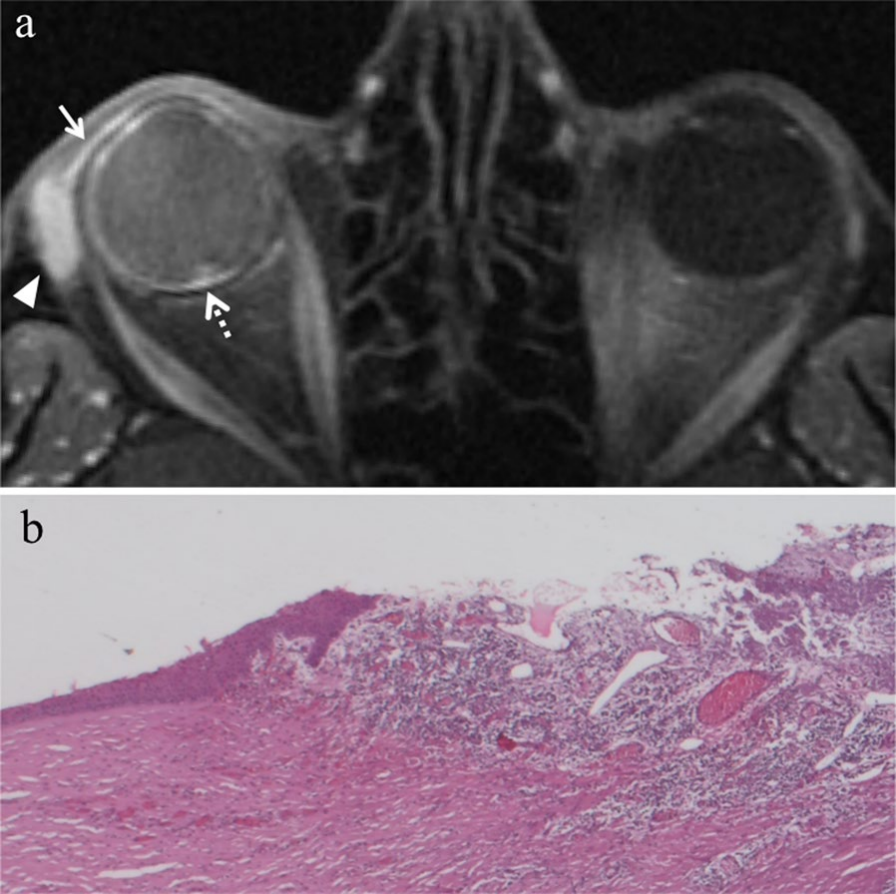
A 58-year-old man with radiation-induced chronic conjunctivitis after proton-beam radiotherapy for choroidal melanoma of the right eye. The same patient as in Fig. 6. **a** Contrast-enhanced fat-suppressed T1-weighted image reveals thickening and marked enhancement of the conjunctiva of the right eye (white arrow). The right lacrimal gland is also enlarged with noticeable enhancement (white arrowhead). Note the residual irradiated tumor along the posterior aspect of the globe (dotted white arrow). **b** Sub-conjunctival inflammatory infiltrate, mainly composed of lymphocytes, ulcerating the overlying stratified squamous epithelium (H&E, original magnification 150×)

Radiation-related dry eye, keratitis and chronic conjunctivitis (Fig. 10) represent complications of both plaque brachytherapy and proton beam radiotherapy and are caused by direct effect of radiations on conjunctival and corneal epithelium as well as by alterations of the tear film induced by radiotherapy. Symptomatic treatment with topical lubricants is recommended [34, 51].

### Lens

**Fig. 11:**
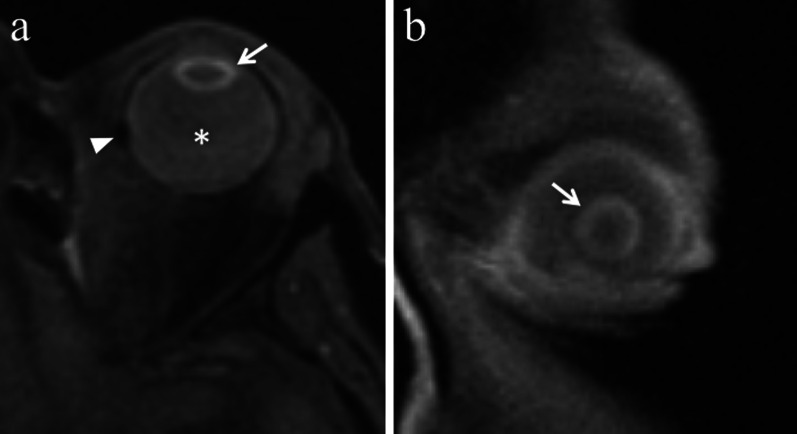
A 65-year-old man with radiation-induced cataract after proton-beam radiotherapy for choroidal melanoma of the left eye. **a** Axial and **b** coronal fat-supressed T1-weighted images show a subcapsular peripheral hyperintense rim of the left lens (white arrows), consistent with radiation-induced cataract. Note the slight hyperintensity of the vitreous body (white asterisk in **a**) due to intraocular inflammation. A small metal artefact (white arrowhead in **a**), due tantalum clip, is detectable along the medial aspect of the sclera

The lens represents the most radiosensitive tissue of the eye [23]. At doses > 10 Gy, radiations cause deformation of lens fibres and subcapsular storage of debris, resulting in cataract formation [4]. At MRI, the lens may lost its typical homogeneous intermediate signal intensity on T1-weighted images and may demonstrate a subcapsular peripheral hyperintense rim (Fig. 11). Radiation-related cataract usually appears in the form of posterior capsular opacities; nevertheless, in case of large tumors and high radiation dose to the lens a total white cataract may occur. In this latter case, it is always preferable to perform cataract surgery, even in the absence of a real visual benefit, in order to make it possible fundus examination [23].

### Vitreous hemorrhage

**Fig. 12:**
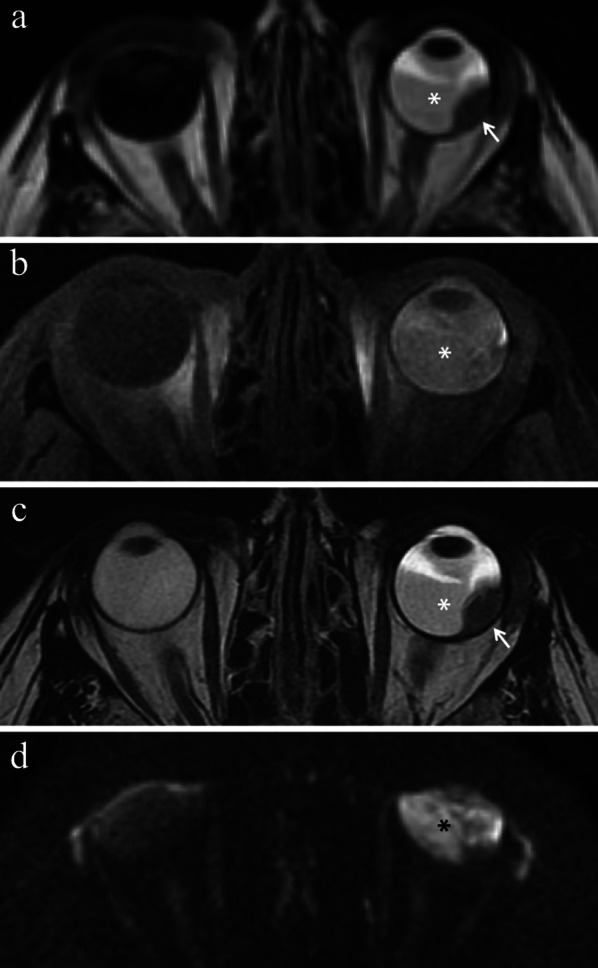
A 75-year-old woman with radiation-induced vitreous hemorrhage after proton-beam radiotherapy for choroidal melanoma of the left eye. The patient underwent secondary enucleation one year after radiotherapy because of painful eye. Axial (**a**) T2-weighted FLAIR (from brain MRI scan), **b** fat-suppressed T1-weighted, **c** T2-weighted turbo spin-echo and (**d**) DW (b = 1000 s/mm^2^) images. The anterior chamber and the vitreous body of the left eye demonstrate inhomogeneous high signal intensity on T2-weighted FLAIR and T1-weighted images (white asterisks). On T2-weighted image, an intraocular fluid–fluid level, with relative hypointensity of the declivous portion (white asterisk), is recognizable within the left globe. Note the difference with the physiological water-like signal intensity of the contralateral eye. On DW image, the left vitreous body exhibits restricted diffusion with high signal intensity (black asterisk). The findings are consistent with extensive vitreous hemorrhage. A dome-shaped intraocular lesion (white arrows in **a** and **c**) is recognizable along the lateral aspect of the left globe

Vitreous hemorrhage is more likely after brachytherapy (Figs. 7b and 12) than after proton-beam radiotherapy. It may resolve unbidden in a matter of weeks; however, more rarely it may recur as well. In this latter case, vitrectomy or even enucleation can be required [23].

### Retina

After radiotherapy, the retinal pigment epithelium shows diffuse signs of fibrous metaplasia [41], whereas photoreceptors are degenerated [31]. Retinal circulation subject to radiations demonstrates similar histopathological alterations to that observed in neoplastic vasculature. As a result of radiation exposure, the loss of endothelial cells of retinal capillaries leads to focal capillary occlusion with the development of dilated capillary collaterals characterized by microaneurysms and telangiectasia. The resulting retinal ischemia produces neovascularization, retinal and vitreous hemorrhage, macular edema and exudation with retinal detachment, retinal degeneration and hyalinization; the aforementioned factors characterize radiation-induced retinopathy [4, 34, 35, 42].

High radiation dose causes a rise in capillary permeability that, in turn, may determine the onset of an acute exudative retinal detachment; this latter generally resolves in a few weeks. On the other hand, late exudative retinal detachment may manifest as a consequence of delayed radiation-related vasculopathy and can be associated with rubeosis and secondary glaucoma (‘toxic tumor syndrome’) [4].

### Iris

The ischemia produced by radiation retinopathy determines overgrowth of iris vessels. The latter clinically manifests in the form of rubeosis iridis [34]. Rubeosis iridis results in secondary (neovascular) refractory glaucoma that can determine blind and painful eye, representing one of the main indications for enucleation [22, 34].

### Toxic tumor syndrome

**Fig. 13:**
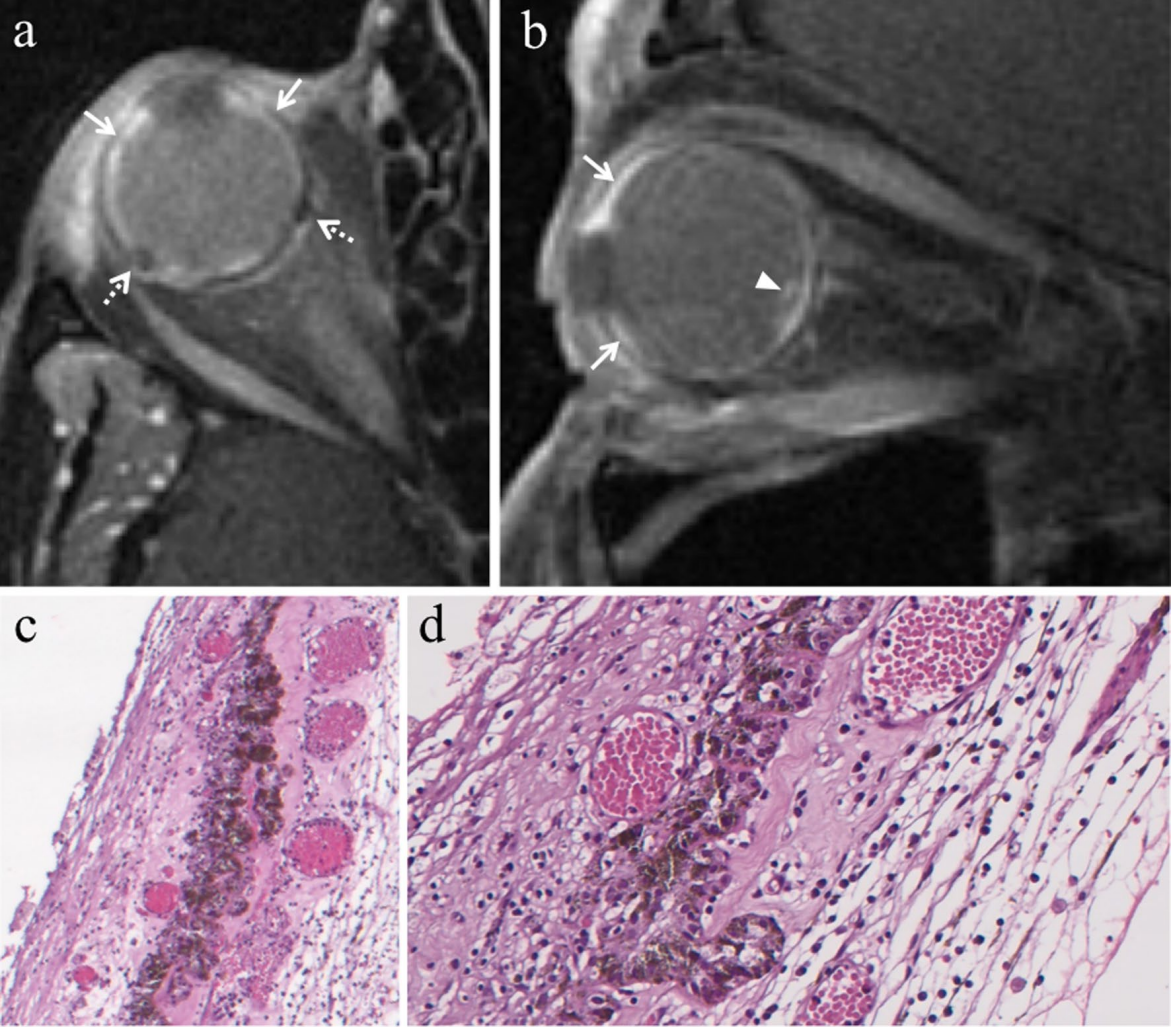
A 58-year-old man with neovascular glaucoma after proton-beam radiotherapy for choroidal melanoma of the right eye. The same patient as in Fig. 6. **a** Axial and (**b**) sagittal contrast-enhanced fat-supressed T1-weighted images display marked enhancement of the ciliary body and the anterior portion of the choroid (white arrows), a finding consistent with anterior segment neovascularization leading to neovascular glaucoma. Note the residual irradiated tumor along the posterior aspect of the globe (white arrowhead in** b**). Small hypointense artefacts produced by the tantalum clips are appreciable along the posterior edge of the sclera (white dotted arrows in **a**). **c** Histological examination showing a florid angiogenic process surrounding a ciliary body (H&E, original magnification 150×). **d** At higher magnification, angiogenesis consisting of numerous congested end ectatic vessels is well documented (H&E, original magnification 400×)

Toxic tumor syndrome is related to the persistence of the irradiated tumor and is characterized by radiation-induced retinal detachment, rubeosis and neovascular glaucoma. The pathogenesis of toxic tumor syndrome has been clarified. The residual scar resulting from tumor irradiation may synthesize proinflammatory cytokines and vascular endothelial growth factor (VEGF) that cause intraocular inflammation and anterior segment neovascularization (Fig. 13); the radiation-induced ischemic alterations of the retina contribute to neovascularization too. These alterations lead to neovascular glaucoma that generally manifests between 2 and 5 years after radiotherapy and represents one of the primary causes of enucleation [23]. Toxic tumor syndrome is more likely in case of bulky tumors, can be treated with intravitreal steroids or antiangiogenic inhibitors (anti-VEGF) and can be prevented by performing tumor resection or transpupillary thermotherapy on the residual tumor scar after proton beam therapy [23, 25, 34, 52, 53].

### Choroid

Radiation-related alterations are mainly vascular and encompass telangiectatic and microaneurysmal vascular dilation, vascular congestion, as well as neoangiogenesis (Fig. 6b) [4, 31].

### Optic nerve

**Fig. 14:**
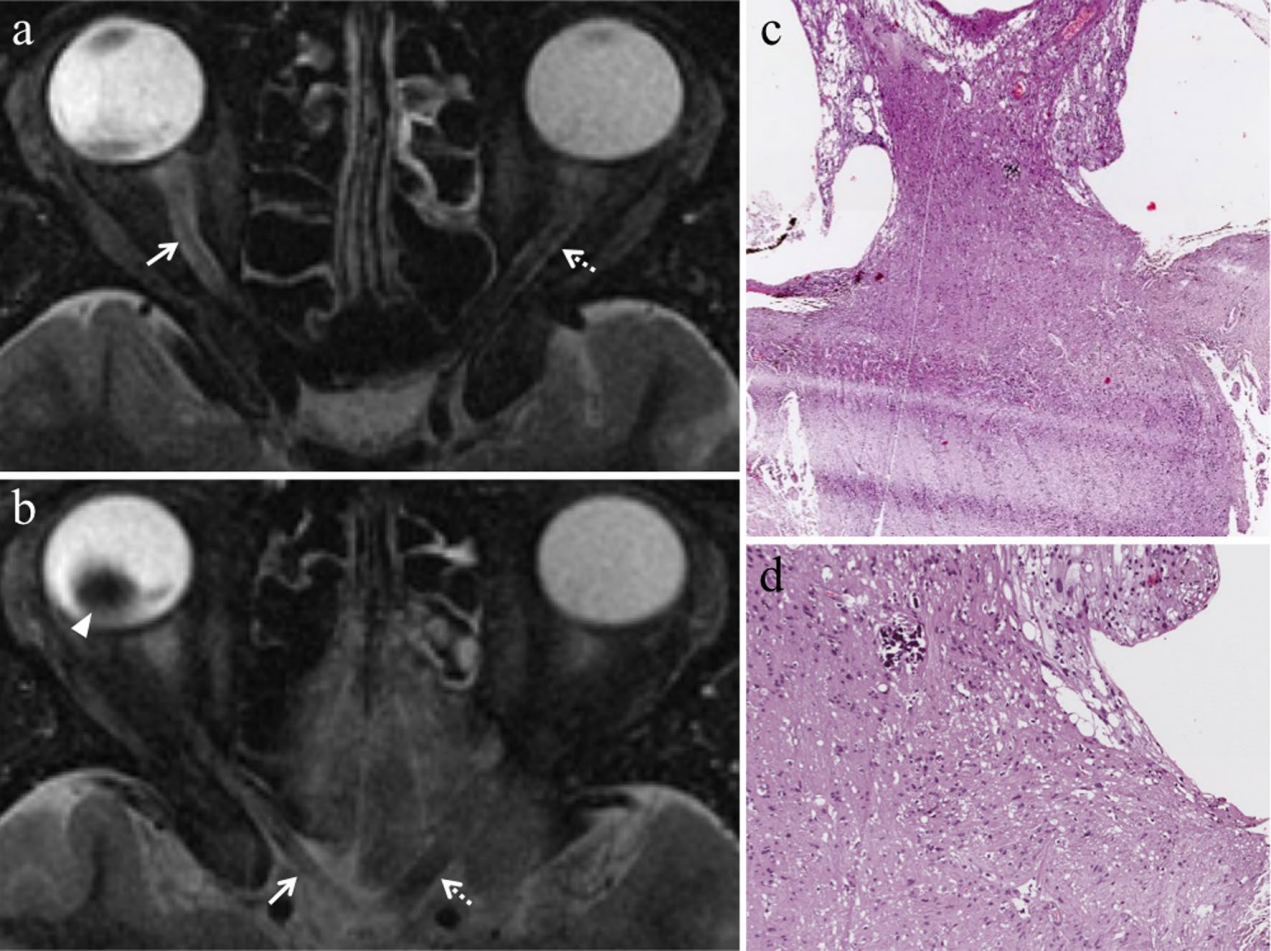
A 37-year-old man with radiation-induced optic neuropathy after proton-beam radiotherapy for choroidal melanoma of the right eye. The same patient as in Fig. 9. **a, b** Axial T2-weighted turbo spin-echo STIR images show an intraocular lesion (white arrowhead in **b**) along the posterosuperior aspect of the globe. The right optic nerve appears thinned and slightly hyperintense (white arrows) compared to the contralateral one (white dotted arrows).** c** Histological examination showing the optic nerve tissue with diffuse degenerative changes: a diffuse fibrosis, extensively dissociating the nerve fibres is evident (H&E, original magnification 50×). **d** Histological detail showing the presence of fibrosis, microcalcifications and microcystic changes, affecting the optic nerve and consisting with a radiotherapy-related degenerative process (H&E, original magnification 150×)

Radiation-induced optic neuropathy is caused by a radiation dose > 50 Gy and clinically manifests with significant visual loss, generally 1.5–2 years after radiotherapy. Believed physiopathogical mechanisms include a direct neuropathic effects of radiation as well as radiation-induced vasculopathy. Histopathologically, the optic nerve demonstrates sectoral or diffuse atrophy with areas of demyelination, neuronal degeneration, necrosis and lymphocytic infiltrates [4, 23, 42]. At MRI, the optic nerve may appear thinned and slightly hyperintense on T2-weighted images compared to the contralateral one (Fig. 14).

### Lacrimal complications

**Fig. 15:**
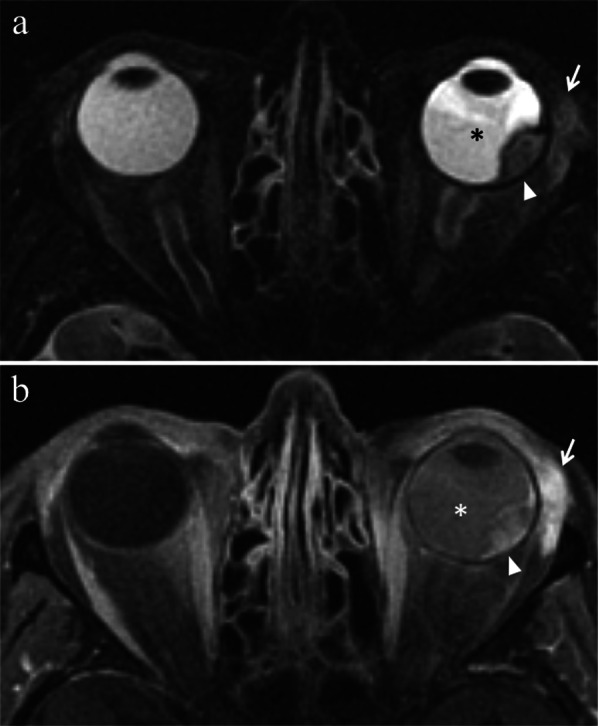
A 75-year-old woman with inflammatory swelling of the lacrimal gland after proton-beam radiotherapy for choroidal melanoma of the left eye. The same patient as in Fig. 12. Axial **a** T2-weighted turbo spin-echo STIR and **b** contrast-enhanced fat-suppressed T1-weighted images demonstrate an enlarged left lacrimal gland with marked enhancement (white arrows). A dome-shaped intraocular lesion (white arrowheads) is recognizable along the lateral aspect (temporal area) of left globe, adjacent to the lacrimal gland. Note the inhomogeneous signal intensity of the left vitreous body on T2- and T1-weighted sequences (asterisks), a finding consistent with vitreous hemorrhage

When treating uveal melanomas of the nasal area with proton-beam radiotherapy, medial canthus and puncta may lay in the radiation field. Radiation-related inflammation may determine canaliculitis, intraluminal adhesion, scarring and obstruction of the lacrimal drainage system, eventually resulting in intractable epiphora [51]. On the other hand, when the lacrimal gland is included in the radiation field (uveal melanomas of the temporal area), gland atrophy with keratoconjunctivitis sicca may occur, usually preceded by an inflammatory swelling of the gland at an early stage (Fig. 15) [25].

### Extraocular muscles and periocular tissues

During treatment with plaque brachytherapy, extraocular muscles may be exposed to a significant radiation dose. Muscles can show ultrastructural alterations consisting in decrease in muscular fibres and increase in fibroblasts and collagen. These disorders may impair extraocular muscle function with the onset of transient diplopia [34].

**Fig. 16:**
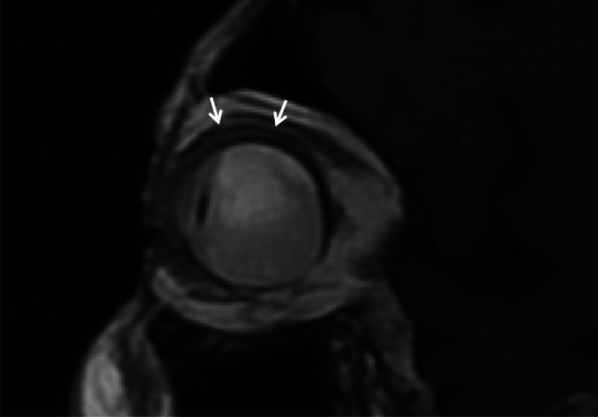
An 82-year-old man with radiation-induced periocular fibrotic adhesions after brachytherapy for choroidal melanoma of the right eye. The same patient as in Fig. 7. Sagittal T1-weighted image shows soft tissue thickening along the superior outer edge of the sclera (white arrows), a finding consistent with fibrotic adhesions

At the level of periocular tissues, radiotherapy and radiation-related inflammatory response may determine dense fibrotic adhesions that can make it difficult the surgical intervention when secondary enucleation is required (Fig. 16) [32]. As proof of this, Pham et al. found that, because of radiation-related scar tissue formation and fibrosis, secondary enucleation conducted after I-125 plaque brachytherapy was technically more challenging and required longer operative time than enucleation performed primarily [54].

### Cosmetic side effects

Cosmetic issues after irradiation encompass orbital hypoplasia, hyperpigmentation of the skin, radiation dermatitis and soft tissue fibrosis [51].

Irradiation of the eyelid during proton beam radiotherapy can induce acute burn, then resulting in a depigmented scar and eyelash loss; squamous metaplasia of the tarsal conjunctiva and keratinization of the mucocutaneous junction in correspondence of the superior lid margin may develop as well. To avoid these complications, proton beam radiotherapy should be performed through the closed eye [25, 52].

### Sympathetic ophthalmia (SO)

SO is characterized by an autoimmune inflammatory disease usually occurring as a consequence of penetrating ocular injury or intraocular surgery. The pathogenesis of SO lies on an autosensitization of blood and vitreous T lymphocytes against an antigenic protein from the uvea or retina. In patients with uveal melanoma treated with proton beam radiotherapy, SO involving the untreated eye has been reported as a rare complication with an incidence of 0.06% [55]. In this case, the radiation-related disruption of uveal tissue should induce expression of melanocytic antigens that, in turn, should be responsible for the T-cell response specific for melanocytes implicated in the onset of the autoimmune process. Although exceedingly rare, SO following proton beam therapy must be timeously recognized and treated with corticosteroids, immunosuppressive drugs and intravitreous anti-VEGF injections, since if misdiagnosed may result in blindness [55].

## Post treatment follow-up

Regardless of the kind of radiotherapy employed, to regularly monitor patients undergoing globe-retaining therapies is mandatory in order to early detect and treat both recurrences and treatment-related complications. Basically, after radiotherapy, patients should be checked initially every 3–6 months for 2 years and subsequently every 6–12 months. At each follow-up, the ocular oncologist has to perform a full ocular examination completed with local tumor assessment.

Systemic surveillance has the purpose of identifying metastatic disease as early as possible; nevertheless, there is no universally accepted consensus on the time interval and on the type of imaging examinations that should be performed. Liver US (performed every 6 months), computed tomography (of the head, chest, abdomen and pelvis), magnetic resonance imaging (of the upper abdomen) and whole body positron emission tomography/computed tomography (PET/CT) are employed in the staging of uveal melanoma both at baseline and in the follow-up [13, 14, 23, 56]. It is conceivable that the recent advances in the field of genetic prognostication and the possibility to stratify uveal melanomas into molecular classes with different metastatic risk may influence also the systemic surveillance of this neoplastic disease [57, 58].

### Role of liver-targeted therapies and interventional radiology

Up to 50% of patients affected by uveal melanoma develop metastases. These latter have an elective tropism for the hepatic parenchyma, so that the liver represents the primary site of metastatization in more than 90% of cases and about 50% of these patients have purely liver metastases [59–61]. Without any therapy, the median overall survival of patients affected by uveal melanoma with hepatic metastases is < 6 months [62]. Therefore, the therapeutic management of liver metastases from uveal melanoma represents, even today, an unresolved issue. Although potentially curative, surgical resection of liver metastases is reserved for thoroughly selected cases (based on lesion distribution and size) and is suitable for less than 10% of patients [60, 61, 63]; at the same time, currently, no systemic treatment has yet proved to be really effective for metastatic uveal melanoma. Thus, in this scenario, regional therapies have been employed in an effort to stabilize hepatic metastases and to arrest the disease progression [64].

In recent years, there has been a gradual transition from open surgical techniques to minimally invasive approaches. The latter encompass hepatic intra-arterial chemotherapy, hepatic transarterial chemoembolization, hepatic perfusions and radioembolization. Such liver-targeted therapies have a twofold advantage: (1) to deliver a concentrated dose of chemotherapeutic agents to both radiologically appreciable and occult lesions, (2) to reduce the drug-related systemic toxicity.

#### Hepatic intra-arterial chemotherapy

Hepatic intra-arterial (HIA) chemotherapy involves the administration of different chemotherapeutic agents (fotemustine, melphalan, cisplatin) by the intra-arterial route through temporary or implantable catheters placed into the hepatic artery surgically or radiologically (via transfemoral approach); the catheters may be connected with a subcutaneous infusion pump. Median overall survival ranges between 9 and 21 months [59, 65]. Procedure-related complications range between 0 and 17% and encompass: thrombosis, infection, leakage and catheter displacement [59].

#### Hepatic transarterial chemoembolization

Hepatic transarterial chemoembolization (TACE) manages to improve the dwell time of chemotherapy agents and to cause select ischemia in the neoplastic tissue, at the same time. The procedure involves the transarterial administration of chemotherapeutic agents followed by an embolic agent. Median overall survival is about 10 months [59, 65]; nevertheless, when interpreting the data, it should be considered that this interventional technique is often employed as a second-line treatment [65].

#### Isolated hepatic perfusion

Isolated hepatic perfusion (IHP) is an operative procedure implying the surgical isolation of the vascular supply to the hepatic parenchyma in order to deliver high dose of chemotherapeutic agents.

In spite of encouraging results in terms of overall tumor response rates (33–62%) and median overall survival duration (10–24 months) [59, 60, 66], IHP has various drawbacks, since it is nonrepeatable, time-consuming (7–8 h) and burdened by high morbidity and protracted hospitalization [59].

#### Percutaneous hepatic perfusion

Percutaneous hepatic perfusion (PHP) is an easier and repeatable nonsurgical alternative to IHP, implying the administration of the chemotherapeutic agent through the hepatic artery and the isolation of hepatic venous flow through a double-balloon catheter placed in the inferior vena cava. The hepatic venous blood is filtered, via an extracorporeal filtration system, which eliminate the drug, before being re-injected into the systemic circulation. Under this system, it is possible to increase the dose of chemotherapeutic agent to the neoplastic tissue and concurrently to cut down drug-induced systemic toxicity [59, 60, 63, 67]. A median overall survival of 27.4 months has been recently reported in a single-center study [67].

#### Localized radioembolization

Localized radioembolization of branches of the hepatic artery employing yttrium-90 (^90^Y) microspheres can be used as a first-line treatment or a second-line treatment in patients no longer responding to previous local or systemic therapies. This procedure allows to deliver high radiation dose within tumor tissue with relative protection of the surrounding parenchyma, since radioactive microspheres, due to their small size, mainly localize within neoplastic microcirculation [60, 65, 68]. Activity delivered to hepatic parenchyma is limited to ≤ 35 Gy in order to reduce the risk of radiation-induced disease and treatment-related toxicity. Median overall survival is about 19 months [61].

Owing to the continuous advances of liver-targeted therapies, the interventional radiologists have gained importance in the clinical management of patients with uveal melanoma. However, we must not forget that the success of local therapies is linked to the extent of the metastatic involvement of the hepatic parenchyma which represents the main prognostic factor [59]. Therefore, the choice to perform a regional treatment should be carefully evaluated under interdisciplinary consensus.

## Conclusions

The diagnostic and therapeutic management of uveal melanoma requires a multidisciplinary approach involving the ocular oncologist, radiologist, radiation oncologist, pathologist and medical physicist. Owing to the advances of diagnostic imaging and the improvements of treatment methods, the indications of radiotherapy techniques undergo a continuous and significant updating and should be therefore intended as a constantly evolving entity. For this reason, the treatment strategy of uveal melanoma should be personalized, keeping into account tumor-related, patient-related and center-related factors. Especially in case of large tumors, to combine different treatment techniques is often necessary in order to increase the possibility of globe preservation and to improve patients’ quality of life. Patients should be closely followed up after any kind of treatment in order to promptly detect and treat possible recurrences as well as the different complications, for some of which MR imaging is particularly suitable. Lastly, when it comes to therapy of uveal melanoma it is important to remember treating not just the neoplasm, but first and foremost the patient; this latter, in addition to clinical and instrumental follow-up, should also be supported through proper psychological care.

## Data Availability

Data sharing is not applicable to this article as no new data were created or analyzed in this study.

## Abbreviations

ATP: Adenosine triphosphate
COMS: Collaborative Ocular Melanoma Study
CT: Computed tomography
FLAIR: Fluid-attenuated inversion recovery
HIA: Hepatic intra-arterial
IHP: Isolated hepatic perfusion
MRI: Magnetic resonance imaging
PET/CT: Positron emission tomography/computed tomography
PHP: Percutaneous hepatic perfusion
SO: Sympathetic ophthalmia
STIR: Short inversion time inversion recovery
TACE: Hepatic transarterial chemoembolization
TSE: Turbo spin-echo
TTT: Transpupillary thermal therapy
VEGF: Vascular endothelial growth factor
